# The Leukemia-Associated Fusion Protein MN1-TEL Blocks TEL-Specific Recognition Sequences

**DOI:** 10.1371/journal.pone.0046085

**Published:** 2012-09-26

**Authors:** W. Martijn ter Haar, Magda A. Meester-Smoor, Karel H. M. van Wely, Claudia C. M. M. Schot, Marjolein J. F. W. Janssen, Bart Geverts, Jacqueline Bonten, Gerard C. Grosveld, Adriaan B. Houtsmuller, Ellen C. Zwarthoff

**Affiliations:** 1 Department of Pathology, Erasmus MC, Rotterdam, The Netherlands; 2 Department of Genetics and Tumor Cell Biology, St Jude Children’s Research Hospital, Memphis, Tennessee, United States of America; Emory University, United States of America

## Abstract

The leukemia-associated fusion protein MN1-TEL combines the transcription-activating domains of MN1 with the DNA-binding domain of the transcriptional repressor TEL. Quantitative photobleaching experiments revealed that ∼20% of GFP-tagged MN1 and TEL is transiently immobilised, likely due to indirect or direct DNA binding, since transcription inhibition abolished immobilisation. Interestingly, ∼50% of the MN1-TEL fusion protein was immobile with much longer binding times than unfused MN1 and TEL. MN1-TEL immobilisation was not observed when the TEL DNA-binding domain was disrupted, suggesting that MN1-TEL stably occupies TEL recognition sequences, preventing binding of factors required for proper transcription regulation, which may contribute to leukemogenesis.

## Introduction

The translocation t(12;22) fuses the *MN1* and *TEL* genes and leads to AML. The product of this rare fusion consists of 1259 amino acids (aa) encoded by the first *MN1* exon fused to aa 55-452 of TEL [1].

TEL is a repressor from the ETS family of transcription factors, most of which are, unlike TEL, transcriptional activators [2]. The ETS family consists of over 40 genes and is defined by its conserved DNA-binding domain (DBD). TEL belongs to a subset of ETS proteins that contain a second conserved N-terminal helix-loop-helix (HLH) dimerization domain. Dimerization is important for activation of partner kinases such as ABL1 in the TEL-ABL1 fusion protein [3]. TEL is a partner in many leukemic fusion proteins, where its HLH and sometimes also the central domains are fused. MN1-TEL is considered the prototype of leukemia-associated fusions to which TEL contributes its DBD [4]. The combination of the transactivating domain (TAD) of MN1 with the DBD of TEL is thought to perturb transcriptional repression normally executed by TEL.

MN1 is a protein of 1319 aa and contains an N-terminal transcription-activating domain (TAD). MN1 functions as a transcription cofactor, both inhibitory and stimulatory for retinoic acid receptor/retinoic-X-receptor (RAR-RXR) and vitamin D/RXR-mediated transcription [5], [6], [7], [8]. MN1, acting as a co-activator, was shown to bind transcription co-activators such as p300 and SRC3 and to synergistically stimulate the transcriptional activity of the RAR/RXR heterodimer. High *MN1* expression was shown to be a predictor of poor clinical outcome in AML patients with a normal karyotype [9]. Mice receiving transplants of MN1-overexpressing bone marrow rapidly developed myeloproliferative disease [6], [10]. When co-expressed with the inv(16) *Cbfb-SMMHC* fusion gene full blown leukemia developed, resembling human leukemia caused by this fusion gene which is always accompanied by high expression of the *MN1* gene.

The fusion product MN1-TEL inhibits RAR/RXR-mediated transcription and acts as a dominant-negative mutant of MN1. Compared to MN1, the TAD in MN1-TEL is poorly stimulated by p160 and p300/CBP, indicating that the block of RAR/RXR-mediated transcription by MN1-TEL is caused by dysfunctioning of the TAD, rather than by recruitment of co-repressors [11]. Forced expression of MN1-TEL in mice causes leukemias and lymphoid tumors [12], [13], [14]. We hypothesized that the leukemogenic potential of MN1-TEL can be attributed to two distinct characteristics. First, MN1-TEL can stimulate TEL-responsive genes by binding to ETS elements and thereby interfere with the repressive effect of TEL, and secondly MN1-TEL can act as a dominant-negative mutant of MN1, as it efficiently represses RAR/RXR-mediated transcription even in the presence of MN1.

One of the methods to examine the behavior of transcription factors such as MN1, TEL and MN1-TEL in living cells is fluorescence recovery after photobleaching (FRAP). This method gives insight into the mobility of fluorescently-tagged proteins which provides quantitative information about protein-chromatin interactions since binding to chromatin leads to immobilisation [15]. In the present work we investigated the mobility of MN1, TEL and MN1-TEL proteins to gain more insight in transcription deregulation by MN1-TEL. Our results suggest that a large fraction of the MN1-TEL protein is tightly bound to DNA compared to TEL, which might result in inhibition of TEL function.

## Methods

### Fluorescence Recovery After Photobleaching

NIH3T3 cell lines containing the GFP-tagged protein of interest were seeded on collagen type 1-coated coverslips (BD-Biocoat, Breda, The Netherlands) 48 h prior to FRAP experiments. Protein expression was induced 24 h later by adding mifepristone (Invitrogen) to a final concentration of 0.01 µM. If indicated, transcription was inhibited by alpha-amanitin (Sigma Aldrich, 50 µg/ml, 3 h prior to FRAP) or stimulated by ATRA (1 µM, 24 h prior to FRAP). Experiments were performed on a Zeiss LSM 510 Meta confocal microscope (Zeiss, Jena, Germany). GFP was excited at 488 nm and the emitted fluorescence was detected in the 505-530 nm range. Nuclei with fluorescence levels corresponding to physiologically relevant expression levels were selected for imaging and FRAP at a lateral pixel size of 80 nm. A region of interest (ROI) of 10 pixels wide and spanning the width of the nucleus was set up, preferably in the middle of the nucleus. Thousand scans of 21 ms of the ROI were performed. After 100 scans a bleach pulse of three iterations was given. FRAP data were normalized to pre-bleach values by subtracting the measured background and dividing by the average fluorescence intensity of the 50 points before bleaching again, after subtraction of background.

### FRAP Data Analysis

The model-based analysis of the FRAP data by Monte Carlo simulation was previously described [15], [16], [17].Briefly: raw FRAP curves were normalized to pre-bleach values and the best fitting curve (by ordinary least squares) was picked from a large set of computer-generated FRAP curves in which five parameters representing mobility properties were varied: diffusion rate (ranging from 0.04 to 25 µm^2^/s), and two immobile fraction (ranging from 0–90%), with two different residence times in immobile state (ranging from 0.1 to 300 s). The size of the ellipsoid was based on the experimentally-derived average size of the nuclei. The laser intensity profile used in the simulation of the bleaching step was derived from confocal image stacks of chemically-fixed nuclei containing GFP that were exposed to a stationary laser beam at various intensities and varying exposure times.

### DNA Constructs, Generation of Cell Lines and Transient Transfections

See material S1.

## Results

**Figure 1:**
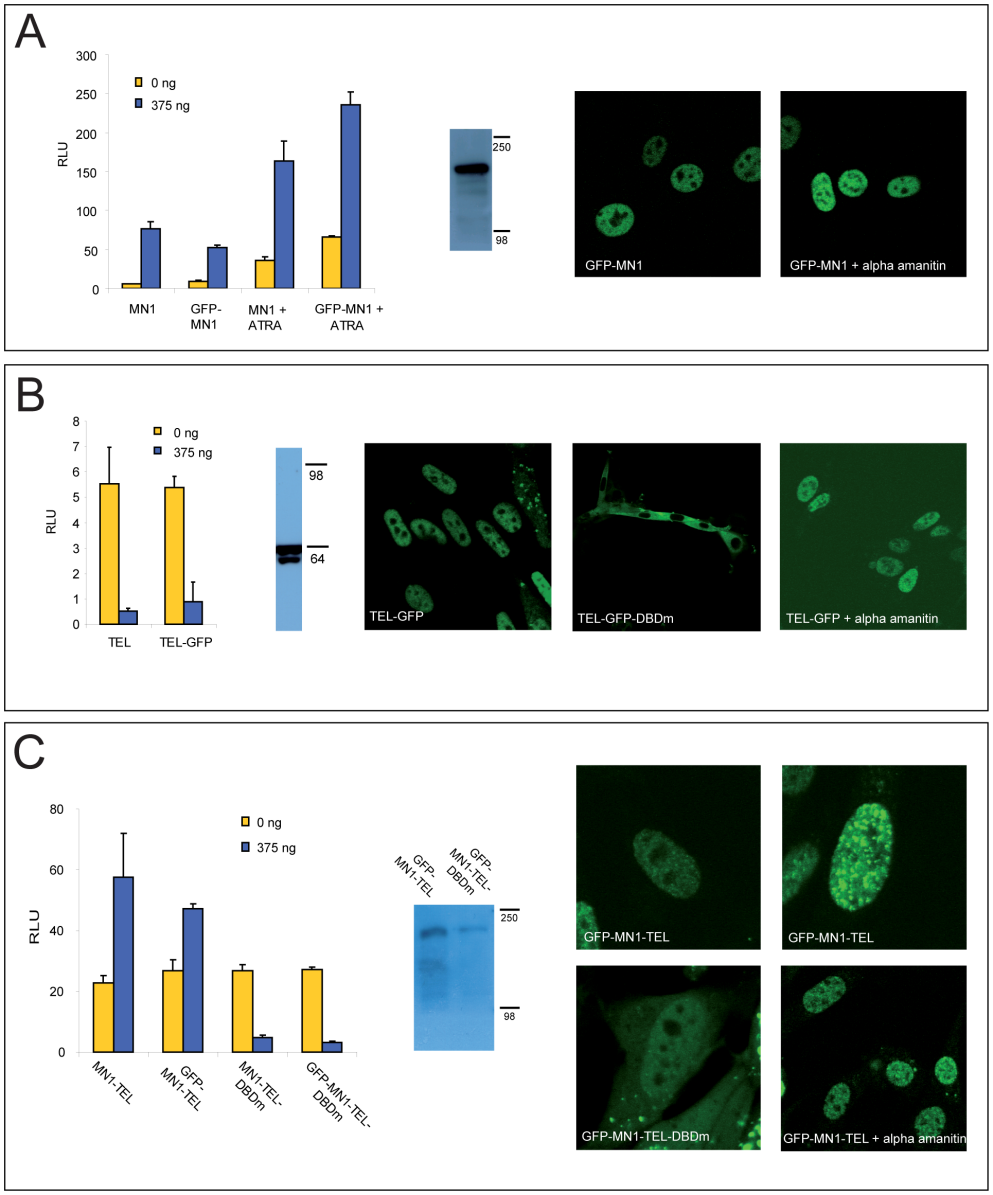
Testing functionality and expression of GFP-tagged MN1, TEL and MN1-TEL. A. GFP-MN1 stimulates transcription from the MSV promoter as efficiently as MN1, both in the presence or absence of 1 uM ATRA (24 h incubation) as tested in with a luciferase assay. Western blot using anti-GFP antibody showing GFP-MN1 protein expression in stably transfected NIH 3T3 cells. GFP-MN1 located to the nucleus, where it was homogeneously distributed, with notable exception of the nucleoli, where no expression could be detected (left photograph). This expression pattern did not change upon alpha-amanitin treatment (right photograph). B. TEL and TEL-GFP are equally able to inhibit gene expression from the MSV promoter. Western blot of a cell lysate of the TEL-GFP-expressing NIH3T3 cell line is stained with anti-GFP and the two isoforms (translation starts at methionine 1 and 43) of TEL are visible. TEL-GFP is mostly nuclear in NIH3T3 cell lines (left photograph). TEL-GFP-DBDm is located in the cytoplasm (middle photograph). Expression pattern of TEL-GFP upon treatment with 50 ug/ml alpha-amanitin for 3 h (right photograph). Both MN1-TEL and GFP-MN1-TEL can weakly stimulate transcription from the MSV promoter, while the DNA-binding mutant is a weak repressor. Western blot with anti-GFP antibody of a lysate of cells expressing GFP-MN1-TEL and GFP-MN1-TEL-DBDm. GFP-MN1-TEL is nuclear, with a tendency to form aggregates upon higher expression levels (upper two photographs). GFP-MN1-TEL-DBDm is both nuclear and cytoplasmic (left lower photograph). Expression pattern of GFP-MN1-TEL did not change upon treatment with 50 ug/ml alpha-amanitin for 3 h (right lower photograph).

To investigate their behaviour in living cells, MN1, TEL and the oncogenic fusion protein MN1-TEL were tagged with EGFP and the effect of the tag on their behaviour was tested. GFP-MN1 and GFP-MN1-TEL were able to stimulate transcription of the MSV promoter at levels similar to their non-GFP tagged versions (Fig. 1A and C). Since GFP tagged directly to either the C- or N-terminus of TEL inhibited TEL-function (data not shown), a spacer was inserted (at the C-terminus) consisting of six glycine-alanine residues, a strategy we previously applied for other transcription factors [18]. TEL-s-GFP was able to repress transcription of the MSV-promoter, similar to wild type TEL (Fig. 1B). The constructs were stably integrated into NIH3T3 cells using the inducible GeneSwitch system. Clones with fluorescence intensities corresponding to wild-type levels were selected and Western Blot analysis showed that the expressed proteins were of the expected size (Fig. 1A, B and C).

**Figure 2:**
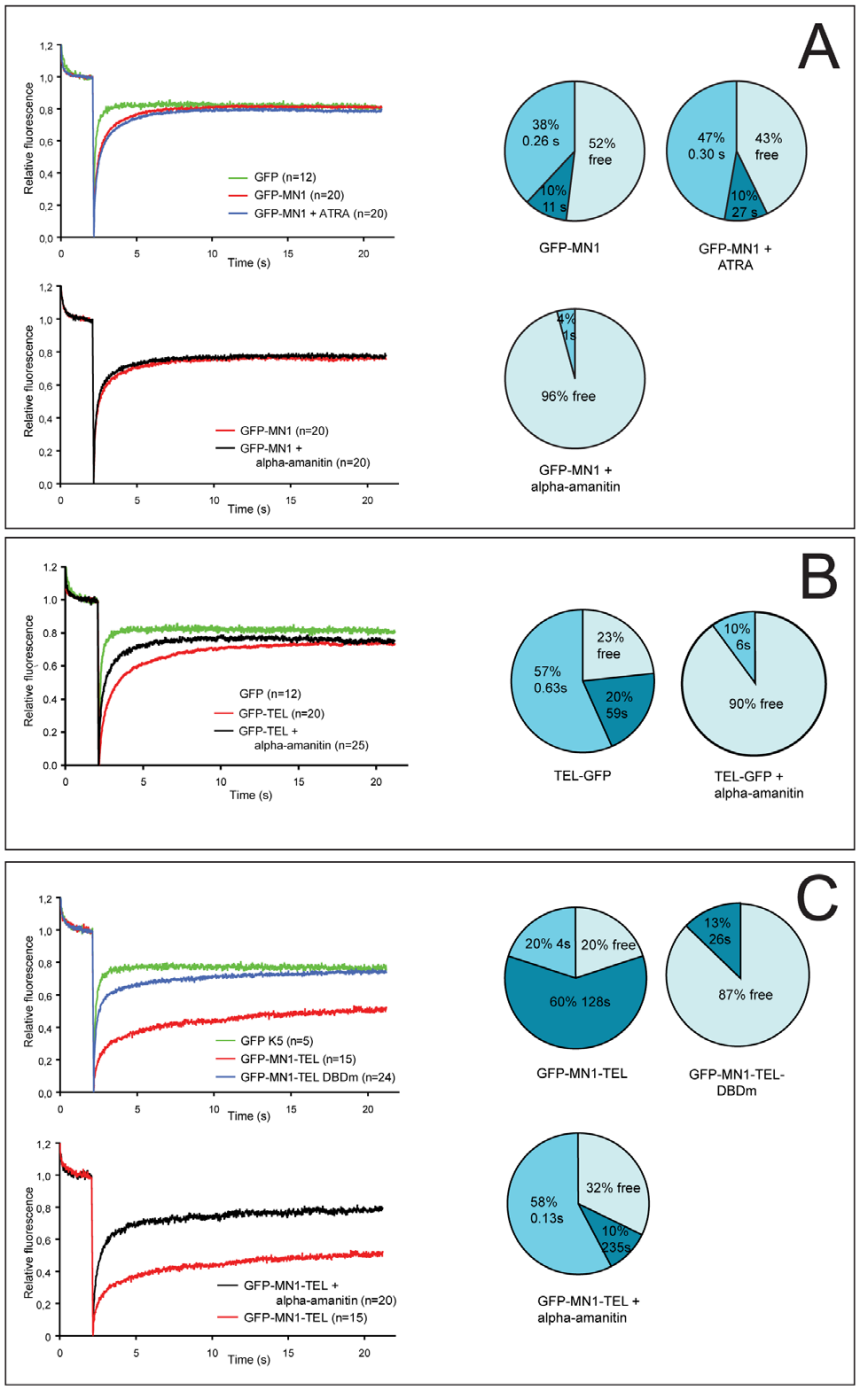
FRAP curves and simulation data. A. GFP-MN1 FRAP curves show the fast recovery of MN1 to levels similar as GFP. In the presence of ATRA the recovery of GFP-MN1 is slightly slower. Alpha-amanitin releases the MN1 protein. Pie diagrams containing simulation data support FRAP curve data. B. TEL-GFP recovered much slower and leveled off to 70% of pre-bleach values. Simulation data calculated the presence of both a long-term bound fraction (20%; 59 s) and a short-term bound fraction (57%; 0.63 s). Alpha amanitin released the TEL-GFP protein as shown by FRAP curve and simulation data. C. GFP-MN1-TEL was largely immobile. A non DNA-binding mutant (DBDm) of GFP-MN1-TEL was mobile and diffused similarly to GFP-MN1. Alpha-amanitin treatment only partly released MN1-TEL. Simulation data calculated that 10% of the protein remains immobile for a long period (235 s).

We then performed FRAP experiments to investigate the behavior of GFP-MN1, GFP-MN1-TEL and TEL-GFP in living cells. As a control we also determined the mobility of free GFP. As expected, the GFP protein by itself rapidly recovered to almost pre-bleach levels (Fig. 2 A, B, and C). TEL-GFP fluorescence recovered much slower than GFP and did not reach the same final level (Fig. 2B), suggesting that a considerable fraction of the protein is immobilised. Quantitative analysis by fitting the experimental curve to curves generated by Monte Carlo simulation confirmed the presence of a ∼20% long-term immobilised fraction of TEL-GFP, with a characteristic binding time of more than a minute (Fig. 2B). In the best fitting scenario, mobile TEL was not completely free but was also involved in very short immobilisation events in the range of 100 ms or less, 75% being involved in these interactions at any moment. The function of TEL in transcription repression, prompted us to examine its mobility when transcription was inhibited by alpha-amanitin [19], [20]. The immobile fraction of TEL-GFP was largely abolished after treatment with alpha-amanitin, strongly suggesting that long-term immobilisation of TEL is due to its involvement in DNA binding.

GFP-MN1 showed a much slower fluorecence recovery than free GFP but to a similar level (Fig. 2A). The experimental FRAP curves were fitted to curves generated by Monte Carlo simulation. Best fits for GFP-MN1 were obtained with simulated curves representing a scenario in which the rate constants of immobilisation and mobilisation are such that ∼10% of the protein is immobile, and individual molecules reside in the immobile state for on average ∼10 seconds (1/K_off_ = 11 s) (Fig. 2A). Since MN1 is a cofactor for retinoic acid (ATRA)-mediated transcription we investigated the effect of stimulating the retinoic acid receptor RAR-RXR. Addition of ATRA lead to a small but reproducible slow down of fluorescence recovery. Interestingly, the immobile fraction did not significantly change, but the residence time in the immobile state was much longer (∼30 seconds). In addition, the fraction involved in short binding events increased. These data suggest that immobilisation is due to binding to immobile transcription complexes, where the residence time in RAR-RXR complexes is longer than in other transcription complexes in which MN1 may be involved (Meester-Smoor et al., 2008). To further investigate this, we determined GFP-MN1 mobility after transcription inhibition by alpha-amanitin, similar to the experiments on TEL mobility. Alpha-amanitin treatment slightly but reproducibly increased the mobility of GFP-MN1 due to almost complete loss of the 10% immobile fraction, in agreement with the hypothesis that immobilisation is due to engagement in transcription (Fig. 2A).

We then applied FRAP to cells expressing the oncogenic fusion protein GFP-MN1-TEL. FRAP revealed that the fusion protein is largely immobile (60%), as fluorescence recovered to only ∼40% of pre-bleach levels (Fig. 2C). Quantitative analysis showed 60% of the GFP-MN1-TEL protein appeared to bind long-term with a characteristic binding time of ∼2 minutes, while another 20% of the protein was predicted to be involved in binding events of on average 4 seconds. Transcription inhibition by alpha-amanitin largely, but not completely abolished the reduced mobility of GFP-MN1-TEL, suggesting that immobilisation is due to binding to TEL recognition sites. To further investigate this, we generated cell lines expressing a non-DNA-binding GFP-MN1-TEL mutant (GFP-MN1-TEL-DBDm), in which the TEL-DBD was dysfunctional. Behaviour and expression of the GFP-tagged mutant were identical to that of the non-tagged protein (Fig. 1C). Unlike the TEL-DBDm, GFP-MN1-TEL-DBDm was largely nuclear. FRAP analysis revealed that GFP-MN1-TEL-DBDm is mobile (Fig. 2C), with diffusion rates similar to GFP-MN1. Importantly, these experiments show that removing the DNA-binding capacity has a significantly stronger effect than transcription inhibition, most likely because transcription inhibition does not fully block DNA-binding of transcription factors, whereas removing the DNA-binding capacity does. This may explain the more limited effect of transcription inhibition on TEL and MN1 mobility. Note that, unfortunately, we were unable to test a TEL non-DNA-binding mutant (DBDm), because nuclear import and DNA binding of TEL is dependent on the mutated amino acids, leading to a fully cytoplasmic localization of TEL-DBDm (Fig. 1B).

**Figure 3:**
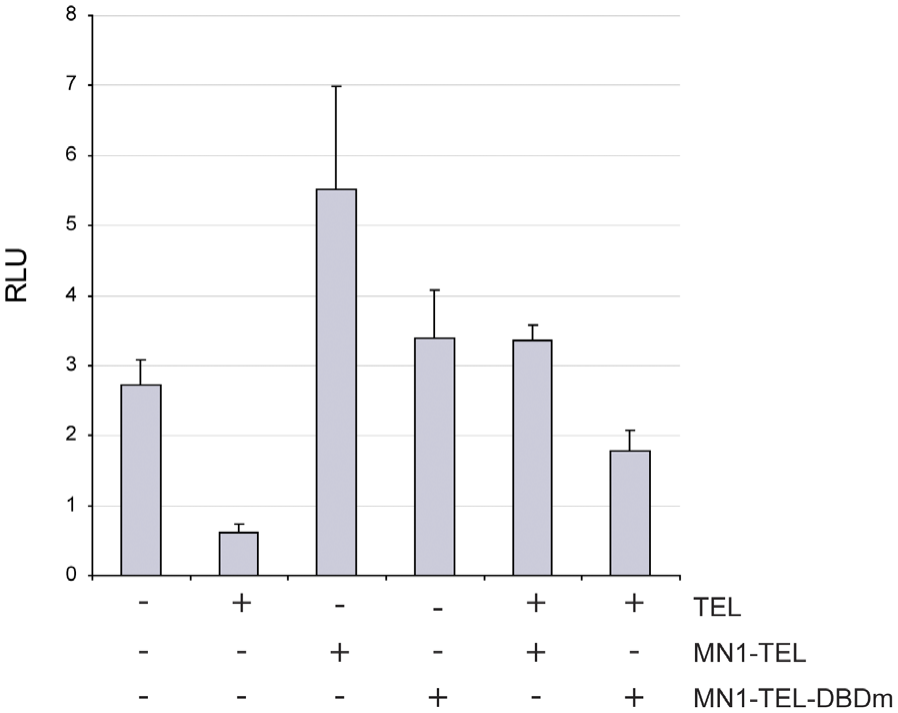
Stromelysin promoter is inhibited by TEL and stimulated by MN1-TEL. Expression and reporter plasmids, 100 ng and 250 ng respectively, were transiently transfected in NIH-3T3 cells. Expression of TEL results in inhibition of stomelysin promoter, whereas MN1-TEL stimulates. The non-DNA-binding mutant of MN1-TEL failed to stimulate. Equal amounts of TEL and MN1-TEL show intermediate promoter activity, indicative of competition of the proteins for the same binding sites.

We then further investigated the hypothesis that MN1-TEL binds TEL-recognition sites, thereby suppressing the repressor function of TEL. The stromelysin promoter is a natural target of TEL and is frequently used to investigate TEL repression [21]. MN1-TEL was able to stimulate the stromelysin promoter, in contrast to TEL, which inhibited promoter activity (Fig. 3). This corroborates the conclusions based on the FRAP experiments above, that both proteins are competing for the same binding sites within the promoter. In addition, adding equal amounts of TEL and MN1-TEL resulted in increased promoter activity, suggesting that MN1-TEL occupies the majority of the promoters. Thus, MN1-TEL not only prevents transcription repression by TEL, but also activates the genes that should be repressed.

## Discussion

In this work we investigated the *in vivo* mobility and DNA-binding behaviour of MN1, TEL and the leukemogenic fusion protein MN1-TEL. The relatively high mobility and small immobilised fraction of MN1 is in concordance with gel filtration experiments of MN1-expressing cells, in which we noticed that over 90% of the protein was found in column fractions corresponding with its monomeric size (unpublished results). MN1 is a protein that affects RAR/RXR-induced gene expression. When we added the RAR ligand ATRA to the medium of cells expressing GFP-MN1, we observed a small decrease in mobility. Monte Carlo simulation suggests that this corresponds to a two- to threefold increase in the characteristic DNA-binding time of the fraction involved in binding events. The remainder of the protein is either free or involved in very transient binding events. Blocking transcription by alpha-amanitin released almost all protein, in accordance with a model where active transcription is necessary to maintain transcription complexes [22] and with data obtained with the transcription factor/repair protein TFIIH, which is also mobilized by alpha-amanitin treatment [23].

Two lines of evidence suggest that immobilisation of TEL and MN1-TEL is due to DNA binding. Firstly, a non-DNA-binding mutant of MN1-TEL does not show a significant immobile fraction. Secondly, the addition of the transcription blocker alpha-amanitin led to mobilization of the proteins. The only other transcriptional regulator known to date with an immobile fraction as profound as that of MN1-TEL is the transcriptional repressor UTF1, which associates with histones in embryonic stem cells [24]. Other proteins known to be involved in long-term silencing processes, such as the heterochromatin-associated protein HP-1 and several members of the polycomb complexes, also are partly immobile, but not to such an extent as MN1-TEL [19], [25].

The mobilization of MN1-TEL and TEL upon addition of alpha-amanitin can be explained in three different ways: first our observations are surprisingly similar to those of HP-1. For HP-1, its increased mobility is suggested by the authors to be the consequence of chromatin decondensation by alpha-amanitin [26]. This suggests that TEL and MN1-TEL may be involved in chromatin condensation, well in line with TEL’s role in long-term repression as proposed by Boccuni et al. [27]. Secondly, it has also been reported that alpha-aminitin can block chromatin decondensation [28], in which case the increased observed mobilities of TEL and MN1-TEL maybe because their activity is no longer required, since after prolonged alpha-amanitin exposure, the majority of chromatin exists in condensed state. Thirdly, since alpha-amanitin has also been shown to lead to degradation of RGB1, the largest subunit op RNA Polymerase II [29], it may be that, irrespective of chromatin state, the mere absence of this most essential transcription factor leads to decay of existing transcription complexes, and prevents formation of new ones. The long period (24 hours) of alpha amanitin-incubation may then finally result in a situation where the majority of transcription factors and related nuclear proteins are mobile, independent from chromatin status.

Irrespective of which of these explanations is correct (they all point to a similar model of TEL function and MN1-TEL dysfunction),it is tempting to the see the long-lasting DNA-interactions of repressors like TEL and UTF1 in the light of the distinct timing of transcription initiation and chromatin remodeling suggested previously by Karpova et al. [30]. These authors provided evidence that the short DNA-interaction times of many transcription factors generally found with FRAP are those associated with actual transcription initiation, while the longer, slower cycling ones found with ChIP (∼15 to 90 minutes) reflect the availability of promoters due to chromatin remodeling rather than actual binding times of individual factors. In view of this model it could be speculated that relatively long DNA-binding times of repressors are related to regulation of the latter process.

The differences in bound fraction suggest that in equilibrium, more MN1-TEL is bound to TEL-recognition sequences than TEL when similar amounts of both proteins are present, because of the longer characteristic binding time of MN1-TEL. Since MN1-TEL can stimulate gene expression from ETS-responsive elements, it could thus induce genes that in the normal situation are inhibited by TEL. Expression of the MN1-TEL fusion protein is under the control of the MN1 promoter. In the hematopoietic system, MN1 expression is confined to the granulocyte-monocyte progenitors (GMP) [10]. TEL expression in these cells has not been investigated, but is not unlikely since expression of TEL is wide spread as judged from expression arrays (http://www.oncomine.org/main/mainx.jsp). It is known that different ETS proteins can bind to the same promoters [31], therefore it could also be that MN1-TEL disrupts the function of other proteins from this family.

The high affinity of MN1-TEL for DNA could be a consequence of the loss of domains in TEL involved in regulation of well-timed release of TEL from the transcription complex. In this regard it is interesting that MN1-TEL misses the first 54 amino acids of TEL as it has been shown that the isoform of TEL which lacks the first 42 amino acids is a more active repressor than the isoform starting at methionine 1 [32]. Sumoylation of lysine 11 in this isoform was shown to interfere with DNA binding [33]. MN1-TEL lacks this residue and may therefore display a larger affinity for DNA than TEL, which is a mixture of both isoforms. Another possibility is that the MN1 moiety in MN1-TEL enables binding of coregulators like p300 and RAC3 [8] that may stabilize the DNA-protein complex and thereby interfere with proper functioning.

Recently, Kawamata et al [34] published FRAP experiments with the PAX5-TEL fusion protein in which it was shown that this protein was also immobile. A fusion between PAX5 and the SAM domain of TEL had similar properties and hence Kawamata et al attribute the immobility to the dimerization/oligomerization properties of the SAM domain. However, we think that this property is not responsible for the immobility of the fusion protein MN1TEL. Although the SAM domain is present in the MN1-TEL fusion protein, we were unable to show binding in a immunoprecipitation assay between MN1TEL and TEL (Buys et al. [35]) and also in binding assays using HA-tagged TEL and *in vitro* transcribed and translated MN1-TEL and TEL, although TEL efficiently bound to itself in the same assays (unpublished results). Moreover, the PAX5 protein contributes its own DNA binding domain to the fusion protein and hence PAX5-TEL encompasses the DNA binding domains of both contributing proteins.

In conclusion, our data suggest two possibilities by which the long-term DNA binding of MN1-TEL may contribute to the leukemogenic process. The first possibility is that MN1-TEL competes with TEL for its cognate binding sites and due to its longer residence time effectively blocks TEL function. This hypothesis requires that TEL is expressed and is functional in GMPs. The second possibility is that MN1-TEL blocks access of other ETS-family members to their cognate binding sites, which are very similar for all family members and perturbs their function.

## Supporting Information

Material S1 (DOC)
