# Supplementary material for: The Leukemia-Associated Fusion Protein MN1-TEL Blocks TEL-Specific Recognition Sequences
Source: PLoS One. 2012 Sep 26;7(9):e46085. doi: 10.1371/journal.pone.0046085 (PMC3458806; doi:10.1371/journal.pone.0046085)
Supplement: Material S1 — (DOC) [file pone.0046085.s001.doc]

**Supplementary material S1**:

*Constructs*  MN1, TEL, MN1-TEL, MN1-TEL-DBDm and GFP-MN1 pcDNA3 and pGENE expression constructs and the pGL2MSV have been described previously (Buijs et al., 1995; Buijs et al., 2000; van Wely et al., 2007). Rat stomelysin promoter cloned in front of luciferase (pGL754TR) was a kind gift of Prof.dr Hiebert (Vanderbilt Univeristy, Nashville, TN, USA). GFP-MN1-TEL was created by exchanging the 5’ *Sfi*I-*Sfu*I fragment of MN1-TEL with that of GFP-MN1. A spacer encoding 6 repeats of the aa alanine and glycine (6xAG) was inserted between TEL and GFP using primers flanked by *Bsm*I and *Kpn*I sites. The pcDNA3-TEL expression construct was digested with *Eco*RI and *Bsm*I and the fragment containing the TEL-coding sequence was isolated. This fragment and the annealed, phosphorylated *Bsm*I-*Kpn*I 6xAG spacer were ligated into *Eco*RI and *Kpn*I sites of the pEGFP-N2 vector (Clontech, Saint-Germain-en-Laye, France), creating a TEL-6xAG-GFP fusion protein. The fusion was recloned into pcDNA3 and pGENE using the *Eco*RI and *Not*I sites.

#### Generation of stable inducible cell lines NIH3T3 mouse cells were obtained from ATCC (ATCC/LGC Standards, Wesel, Germany; ATCC cell line number: CRL-1658) and were cultured in DMEM (Lonza, Breda, the Netherlands) containing 10% FCS (Hyclone, South Logan, USA) and penicillin/streptomycin (Lonza). Stable, inducible NIH3T3 cell lines were created using the GeneSwitch system (Invitrogen, Breda, The Netherlands). Permit: Legislation on genetically modified organisms registration number DMG/RB IG 01-290, The Netherlands. pSwitch was transfected using FuGene (Roche, Almere, The Netherlands) and cells were grown under selection using 100 ug/ml hygromycin (Invitrogen). One of the resulting clones was transfected with the pGene vector containing GFP-MN1, GFP-MN1-TEL, GFP-MN1-TEL-DBDm or TEL-GFP. Cells were grown under selection using 400 μg/ml zeocin (Invitrogen). About 20 of the best growing clones were tested for GFP expression and used for Western blot analysis to check for expression of a protein of the correct size (anti-GFP antibody: No.11814460001, Roche). Clones were cultured under selection using 50 μg/ml hygromycin and 200 μg/ml zeocin.

#### Transient transfections Hep3B cells were cultured in alfa-mem (Lonza) containing 5% FCS, pen/strep and L-glutamine (Lonza). For transient transfections 6.0x10^4^ Hep3B or NIH3T3 cells per well were seeded in 24-well plates. 24h later all-trans retinoic acid (ATRA, Sigma Aldrich, St. Louis, MO, US) was added to a final concentration of 1 uM and transfections were performed, in triplicate, using 3 μl FuGene per μg DNA transfected. The DNA mixture consisted of 25 ng pGL2MSV or 250 ng stomelysin reporter plasmid, 375 ng of pcDNA3-based expression construct or empty pcDNA3 vector as control for MSV-based experiments and 100 ng of expression vector for the stromelysin-based experiment. Total DNA content and the molar amount of CMV promoters were kept equal for each well. 48h after transfection the cells were lysed and luciferase expression was assayed on a Fluoroscan Ascent FL luminometer (Labsystems, Helsinki, Finland).

#### **References:**

#### Buijs, A., Sherr, S., van Baal, S., van Bezouw, S., van der Plas, D., Geurts van Kessel, A., Riegman, P., Lekanne Deprez, R., Zwarthoff, E., Hagemeijer, A. et al. (1995). Translocation (12;22) (p13;q11) in myeloproliferative disorders results in fusion of the ETS-like TEL gene on 12p13 to the MN1 gene on 22q11 [published erratum appears in Oncogene 1995 Aug 17;11(4):809]. Oncogene **10**: 1511-9.

#### Buijs, A., van Rompaey, L., Molijn, A. C., Davis, J. N., Vertegaal, A. C., Potter, M. D., Adams, C., van Baal, S., Zwarthoff, E. C., Roussel, M. F. et al. (2000). The MN1-TEL fusion protein, encoded by the translocation (12;22)(p13;q11) in myeloid leukemia, is a transcription factor with transforming activity. Mol Cell Biol **20**: 9281-93.

#### van Wely, K. H., Meester-Smoor, M. A., Janssen, M. J., Aarnoudse, A. J., Grosveld, G. C. and Zwarthoff, E. C. (2007). The MN1-TEL myeloid leukemia-associated fusion protein has a dominant-negative effect on RAR-RXR-mediated transcription. Oncogene **26**: 5733-40.
